# Filter paper supported nZVI for continuous treatment of simulated dyeing wastewater

**DOI:** 10.1038/s41598-019-47863-5

**Published:** 2019-08-05

**Authors:** Pingping Yu, Haifan Yu, Qisong Sun, Bomou Ma

**Affiliations:** 1 College of Internet of Things Engineering, Department of Electronic Engineering, Jiangnan University, Wuxi, Jiangsu 214122 China; 2 Key Laboratory of Eco-Textiles, Ministry of Education, College of textile and clothing, Jiangnan University, Wuxi, Jiangsu 214122 China; 3 Innovation Center for Textile Science & Technology, Donghua University, Shanghai, 201620 China

**Keywords:** Pollution remediation, Nanoparticles

## Abstract

In this study, polyacrylic acid modified filter paper (FP/PAA) was synthesized by *in-situ* polymerization of acrylic acid, which was used as a matrix to chelate nano-scale zero valent iron (nZVI). The loading content of nZVI in the filter paper reached 24.8%. The fabricated composite FP/PAA/nZVI was characterized by SEM, FT-IR and TGA respectively. Moreover, it was used for the removal of methyl blue and methylene blue as model anionic and cationic dyes. The effect of initial dye concentration on decolorization efficiency was investigated. The results showed that FP/PAA/nZVI enhanced the removal of dye from the simulated dye wastewater and the decolorization efficiency exceeded 95% for the dye solutions lower than 20 mg/L. More importantly, the filter paper supported nZVI realized the continuous treatment of simulated dye wastewater by a simple filtration process. This study hopes to serve as a basis for the application of nZVI in textile wastewater treatment.

## Introduction

In the textile dyeing industry, untreated dye wastewater undoubtedly causes serious environmental problems and deteriorates groundwater quality, which is associated with the health of human and other creatures. Thus, it is necessary to remove the dye from wastewater before they are discharged into the environment. In previous literatures, some methods, including flocculation^1^, fenton oxidation^2^, membrane filtration^3^, adsorption^4^, photochemical^5–7^, ion exchange^8^, electrochemical oxidation^9^ and electrolytic precipitation^10^, had been used for the treatment of dye wastewater. However, the limitations of the respective methods restrict their application, i.e. flocculation and fenton oxidation produce sludge, photochemical forms by-products, electrochemical oxidation involves high cost of electricity and electrolytic precipitation needs long time^11^. Moreover, dye wastewater needs effective treatment to achieve complete mineralization, which is not possible with the available methods^12^. In recent years, the application of nanotechnology in environmental remediation has drawn the attention of researchers. Mohamed *et al*. investigated the adsorption of methyl green using multi-walled carbon nanotubes^13^. Liu *et al*. used 3D graphene to remove water-soluble dye^14^. Wang *et al*. reported the adsorption of azo dye on nanodiamond^15^. In addition, nano-titanium dioxide (nano-TiO_2_), nano-zinc oxide (nano-ZnO), nano-magnesium oxide (nano-MgO) and nano-scale zero valent iron (nZVI) were also studied for dye removal, respectively^16,17^. Compared to other nano-materials, nZVI is inexpensive, easy to prepare, has high reactivity and less toxic effect on humans^11,18^, and have received more focus in recent year. The application of nZVI in removing various contaminants, including nitrates, phenols, heavy metals and pesticides, had been reviewed by references^19,20^. However, nZVI easily aggregate, has rapid sedimentation and oxophilicity due to its magnetism and small size effect^21,22^, which leads to the decrease in the activity of nZVI, resulting in a decrease in the degradation rate of contaminants. Much effort has been made by researchers to solve these problems. Some studies used surfactants and coatings to stabilize nZVI via steric hindrance and electrostatic repulsion, such as sodium oleate and sodium dodecyl sulfate^23^, Tween and cetyltrimethylammonium bromide^24^, polyethylene glycol and polytetrahydrofuran^25^, rhamnolipid and carboxymethyl cellulose^26^. Other studies employed host materials to immobilize nZVI. The reported host materials included PAN^27,28^, cellulose^29^, calcium alginate^30,31^, modified clays^32,33^, clinoptilolite^34^, mesoporous molecular sieves^35^, and porous polymers^36^.

Among all the reported stabilizers, cellulose-based materials were regarded as the best for their low cost, biodegradability and are environmentally benignant. Filter paper (FP), a common laboratory consumable, is composed of oxygen rich cellulose fibers with pores. The architectural structure of FP offers many anchoring sites and inner cavities for trapping nanoparticles. FP embedded with silver nanoparticle^37^, copper nanoparticle^38^, gold nanoparticle^39^, titanium dioxide particle^40^ and nZVI^41^ had been fabricated for water treatment, dye removal, reaction monitoring and water-oil separation, respectively. However, all of them are intermittent and discontinuous. There are no reports about the continuous treatment of pollutions. In this study, nZVI was embedded in polyacrylic acid modified FP (FP/PAA) by a simple procedure and was proposed for dye (including methyl blue and methylene blue) decoloration. The introduction of PAA caused the ferric ion to be firmly anchored in FP. The study investigated the properties of the fabricated FP/PAA/nZVI and its effectiveness on targeted pollutants. Most importantly, the study realized the consecutive treatment of dyeing wastewater and is valuable for industrial application.

## Methods

### Materials and reagents

Whatman qualitative filter paper with diameter 9 cm (525.4 ± 8 mg/piece) was purchased from General Electric Healthcare Company (Hangzhou, China). Ammonium persulfate ((NH_4_)_2_S_2_O_8_, APS), Acrylic acid (AA), ethylene glycol (EG), Ferrous sulfate heptahydrate (FeSO_4_·7H_2_O) and Potassium borohydride (KBH_4_) were supplied by Sinopharm Chemical Reagent Co., Ltd. (Shanghai, China). Methyl blue (M100199-25 g) and Methylene blue (M134389-25 g) were supplied by Aladdin (Shanghai, China). All the reagents were used as received without further purification.

### Fabrication of nZVI imbedded FP/PAA (FP/PAA/nZVI)

In accordance with the reference^27^, FP was firstly modified with polyacrylic acid (PAA) via an *in-situ* polymerization of AA, which produced the carboxylic acid groups and chelates the ferric ion. The solution used for modification was prepared by mixing AA(86.0 g), APS (2.0 g), EG (6.4 g) and deionized water (105.6 g) in 500 ml beaker. FP was immersed in the solution for 20 mins and dried afterward in a vacuum oven at 60 °C for 5 h. APS initiates AA in a polymerization process and EG crosslinks PAA chains to form a stable 3D network structure. The modified FP was thoroughly washed with deionized water to remove unreacted monomers and non-anchored PAA polymers. The obtained FP samples were named as FP/PAA and its weight was increased by 38 mg compared with the original FP. Dried FP/PAA was soaked in 200 ml of 0.2 M FeSO_4_ solution for 12 h to chelate Fe^2+^ and rinsed with deionized water several times, followed by drying in a vacuum oven. The trapped Fe^2+^/ Fe^3+^ FP were reduced with 0.5 M KBH_4_ solution which resulted in the functional FP/PAA/nZVI. The resulting FP/PAA/nZVI was washed, vacuum dried and stored in a desiccator before characterization and decoloration experiments. The loaded nZVI is 185.8 mg, which is about 24.8% of the total weight of FP/PAA/nZVI.

### Characterization

Morphology of the FP samples were observed with S-4800 field emission scanning electron microscope (Hitachi, Japan) with an operating voltage of 5 kV. Fourier transform infrared spectra were obtained with Nicolet iS10 FT-IR (Thermofisher, America) with attenuated total reflection (ATR) accessory. Thermal stability was recorded with 1100SF TGA (Mettler-Toledo, Switzerland) in a flowing nitrogen atmosphere between 50 and 750 °C at a heating rate of 10 °C/min.

### FP/PAA/nZVI for decoloration of dyes

Methyl blue and methylene blue were selected to prepare the simulated dyeing wastewater (SDW). Decoloration experiments were conducted under room temperature in 500 ml beaker containing 200 ml SDW with different initial concentrations (10 mg/L, 15 mg/L, 20 mg/L, 25 mg/L, 30 mg/L). A piece of FP/PAA/nZVI (dry weight was about 749.2 mg, including 185.5 mg nZVI) was totally immersed in the beaker to initiate the reaction. The reactor was sealed with a plastic wrap and stirred under 200 rpm. At a certain time interval, 5 ml SDW was taken to analyze the absorbance using TU-1901 ultraviolet visible spectrophotometer (Beijing Persee, China) at maximum absorption wavelengths, which are 600 and 660 nm for methyl blue and methylene blue respectively. Moreover, the decoloration efficiency was calculated according to the following equation.

$${\rm{Decoloration}}\,{\rm{efficiency}}=\frac{{C}_{0}-{C}_{t}}{{C}_{0}}\times 100 \% $$

Where *C*_0_ is the initial concentration of SDW, *C*_*t*_ is the SDW concentration at time *t*.

## Results and Discussion

### Characterization of FP/PAA/nZVI

**Figure 1:**
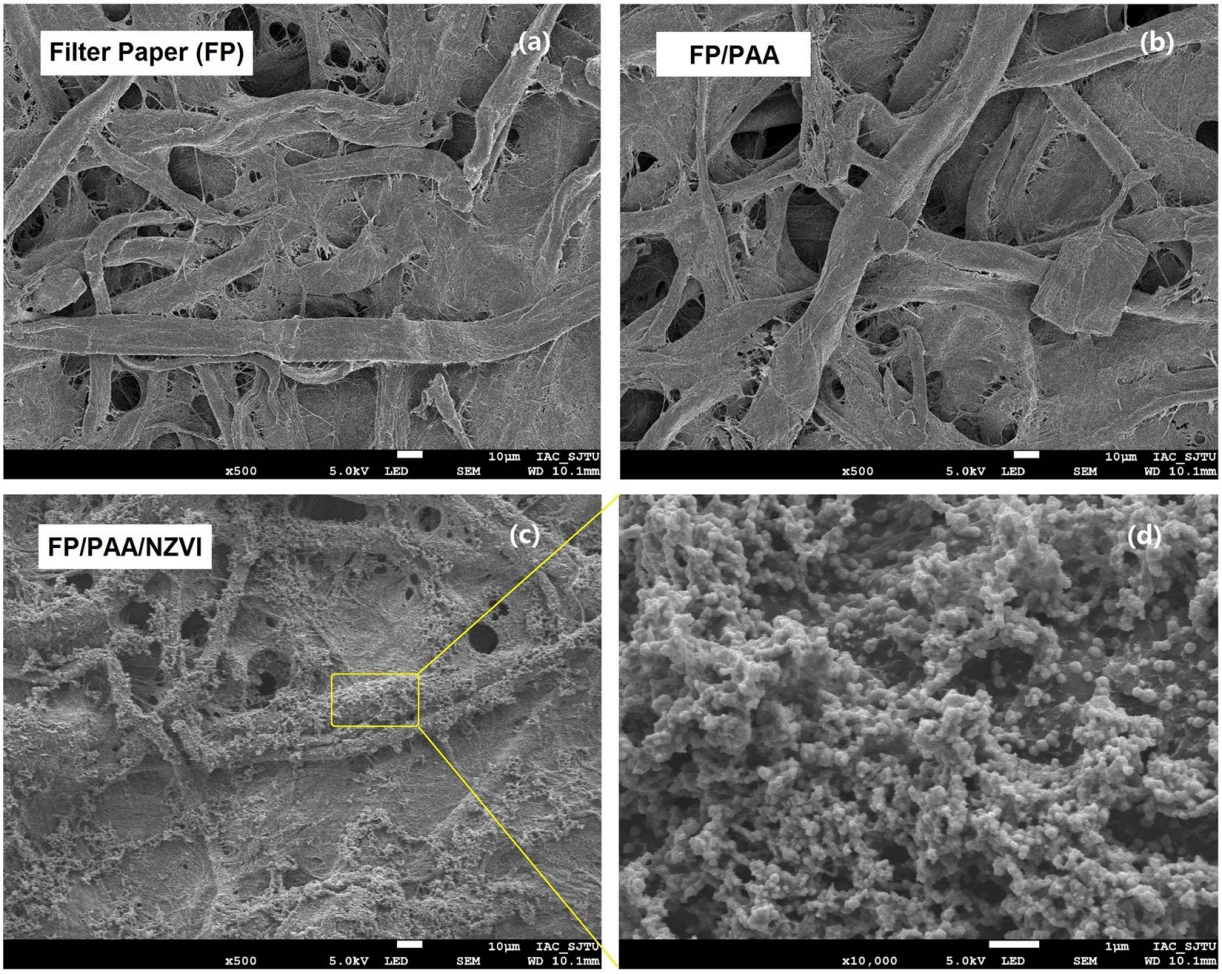
SEM images. (**a**) Pristine filter paper (FP). (**b**) PAA modified filter paper (FP/PAA) by AA *in-situ* polymerization, the PAA was crosslinked by ethylene glycol. (**c**) nZVI was loaded on FP/PAA by chelation of carboxylic acid groups, the loading content is 24.8(wt.)%. (**d**) Magnification of (**c**), morphology of nZVI was presented.

The surface morphology of FP, FP/PAA and FP/PAA/nZVI were observed with FESEM, as shown in Fig. 1, original FP presents a porous microstructure and smooth surface in microfibers, which is similar to the morphology of FP/PAA. However, the result is different from the morphology of PAN/PAA in literature^27^. That is because PAN membrane is hydrophobic. Although it was functionalized by hydrophilic modification, this modification is superficial and cannot penetrate the PAN matrix. This led to AA polymerization on the surface of PAN, resulting in PAA as a coating on the membrane. In this study, filter paper was used as a matrix, which is hydrophilic due to the hydroxyl groups and its porous structure. The AA aqueous solution can infiltrate the FP very well, resulting in a homogeneous FP/PAA matrix and surface, which is beneficial to the subsequent immobilization of nZVI. From the SEM image of FP/PAA/nZVI, it can be seen that a large amount of nZVI particles were anchored on the surface and embedded in the caves of FP. Which is ascribed to the strong chelation between -COOH of FP/PAA and ferric ion from FeSO_4_ solution. The nZVI particles presented a little aggregation, which resulted from the high loading of nZVI in FP/PAA/nZVI. According to the experiment section, the content of nZVI in FP/PAA/nZVI reached 24.8% (185.8 mg), which was determined by the weight method. Compared with other reports^28,41^, the loading content was very high. This ensued from the high concentration and large volume (0.2 M, 200 ml) of FeSO_4_ solution used to modify FP/PAA in the experiment. The loading content can be controlled by varying the concentration and volume of FeSO_4_ solution. Moreover, the high loading content guarantees an excellent decoloration efficiency for SDW.

**Figure 2:**
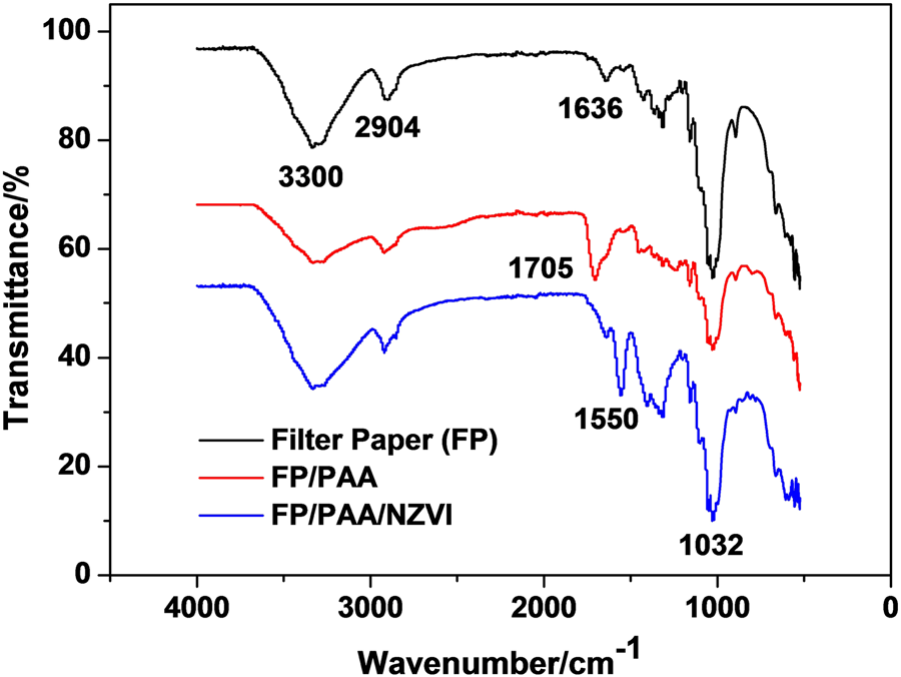
FT-IR spectra of filter paper (FP), FP/PAA and FP/PAA/nZVI from 4000 cm^−1^ to 500 cm^−1^ with attenuated total reflection (ATR) method.

To confirm the presences of carboxylic acid groups and chelation of ferric ion in FP matrix, FT-IR spectra of FP, FP/PAA and FP/PAA/nZVI were conducted. As shown in Fig. 2, the characteristic peaks of FP were observed at 3300 cm^−1^, 2904 cm^−1^, 1636 cm^−1^ and 1032 cm^−1^, which are assigned to the -OH stretching, -CH_2_ stretching, H-O-H stretching and C-O, C-C stretching, respectively^42,43^, and are consistent with characteristic peaks for cellulose fibers. However, when PAA was incorporated into the FP matrix, the peak at 1636 cm^−1^ disappeared and a new peak at 1705 cm^−1^ appeared for FP/PAA, which is assigned to the asymmetric stretching of carboxyl groups^28^. This confirms that an abundant carboxylic acid groups were grafted onto the filter paper and FP was successfully functionalized by PAA, paving way for the next chelation of ferric ion. For FP/PAA/nZVI, the peak at 1705 cm^−1^ disappeared and a new peak at 1550 cm^−1^ appeared, which indicated the chelation between nZVI and carboxylic groups, confirming the loading of nZVI in FP/PAA matrix. It should be noted that, the chelation between nZVI and carboxyl is stronger than the general interaction between nanoparticles and matrix, which ensures the firm immobilization of nZVI in FP.

**Figure 3:**
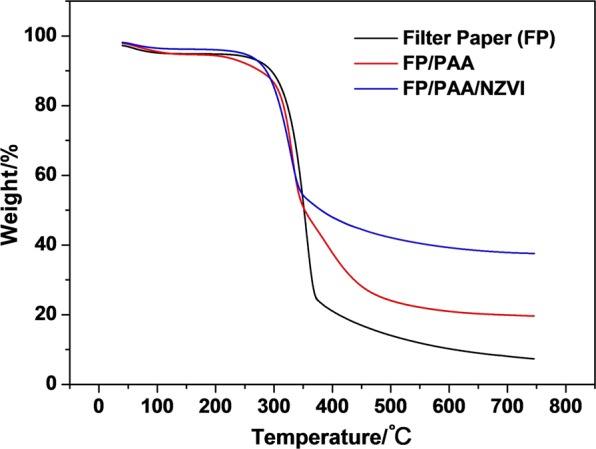
TGA curves of filter paper (FP), FP/PAA and FP/PAA/nZVI from 50 °C to 750 °C under nitrogen atmosphere.

TGA was used to determine the thermal stability of FP, FP/PAA and FP/PAA/nZVI, the results are shown in Fig. 3. All three samples exhibited two stages of decomposition. The first range was from 50 to 150 °C, which corresponds to a mass loss of approximately 5%. The loss was ascribed to the evaporation of water remaining in FP. The range from 200 to 400 °C was attributed to the collapse of cellulose skeleton. However, at the same temperature, the remains of three samples were very different. Specifically, the remains of FP, FP/PAA, FP/PAA/nZVI at 750 °C were stabilized at 7.0%, 19.6% and 37.7%, respectively. The increase from 7.0% to 19.6% resulted from the thermal stability of PAA, which is higher than that of cellulose fibers^44,45^. Since the weight of nZVI was stable under nitrogen atmosphere during the TGA testing condition. The increase from 19.6% to 37.7% was mainly attributed to the development of nZVI on FP/PAA surface (shown in Fig. 1). Therefore, the loading of nZVI in FP/PAA/nZVI could be calculated according to TGA curves using the following equations.

1 $$x+y=100 \% $$

2 $$19.6 \% \ast x+y=37.7 \% $$

Where *x* and *y* refer to the content (wt.%) of FP/PAA and nZVI in FP/PAA/nZVI, respectively. 19.6% and 37.7% refer to the weight remains of FP/PAA and FP/PAA/nZVI at 750 °C, respectively.

The result shows that, the weight content of nZVI in FP/PAA/nZVI is 22.5%, which is consistent with the result for weight method 24.8%. The results from SEM, FT-IR and TGA proved that nano iron particles were immobilized on PAA modified FP by chelation between nZVI and carboxylic groups.

### Decoloration of SDW

**Figure 4:**
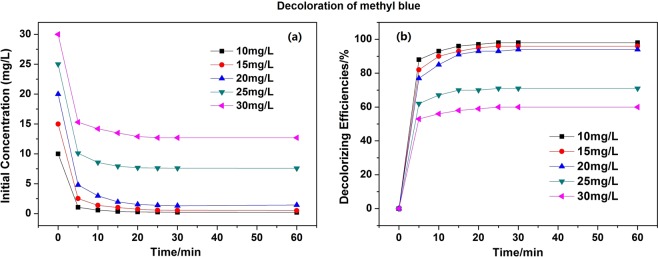
(**a**) Decoloration behavior of FP/PAA/nZVI (content of nZVI is 24.8%) for methyl blue solutions with different initial concentration, 10 mg/L, 15 mg/L, 20 mg/L, 25 mg/L, 30 mg/L. (**b**) Decolorizing efficiency of FP/PAA/nZVI (content of nZVI is 24.8%) for methyl blue solutions with different initial concentration, 10 mg/L, 15 mg/L, 20 mg/L, 25 mg/L, 30 mg/L.

**Figure 5:**
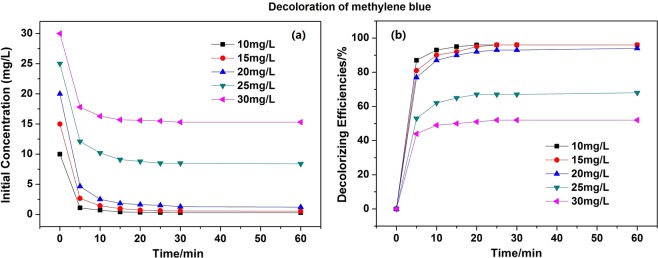
(**a**) Decoloration behavior of FP/PAA/nZVI (content of nZVI is 24.8%) for methylene blue solutions with different initial concentration, 10 mg/L, 15 mg/L, 20 mg/L, 25 mg/L, 30 mg/L. (**b**) Decolorizing efficiency of FP/PAA/nZVI (content of nZVI is 24.8%) for methylene blue solutions with different initial concentration, 10 mg/L, 15 mg/L, 20 mg/L, 25 mg/L, 30 mg/L.

**Figure 6:**
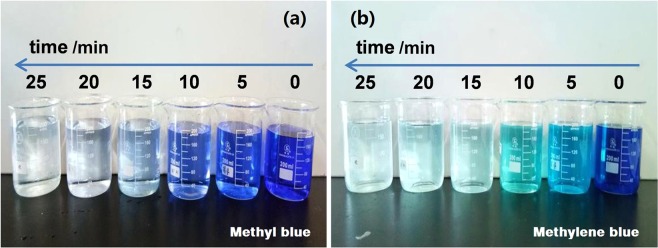
Photograph of treated methyl blue (**a**) and methylene blue (**b**) solutions with time.

**Figure 7:**
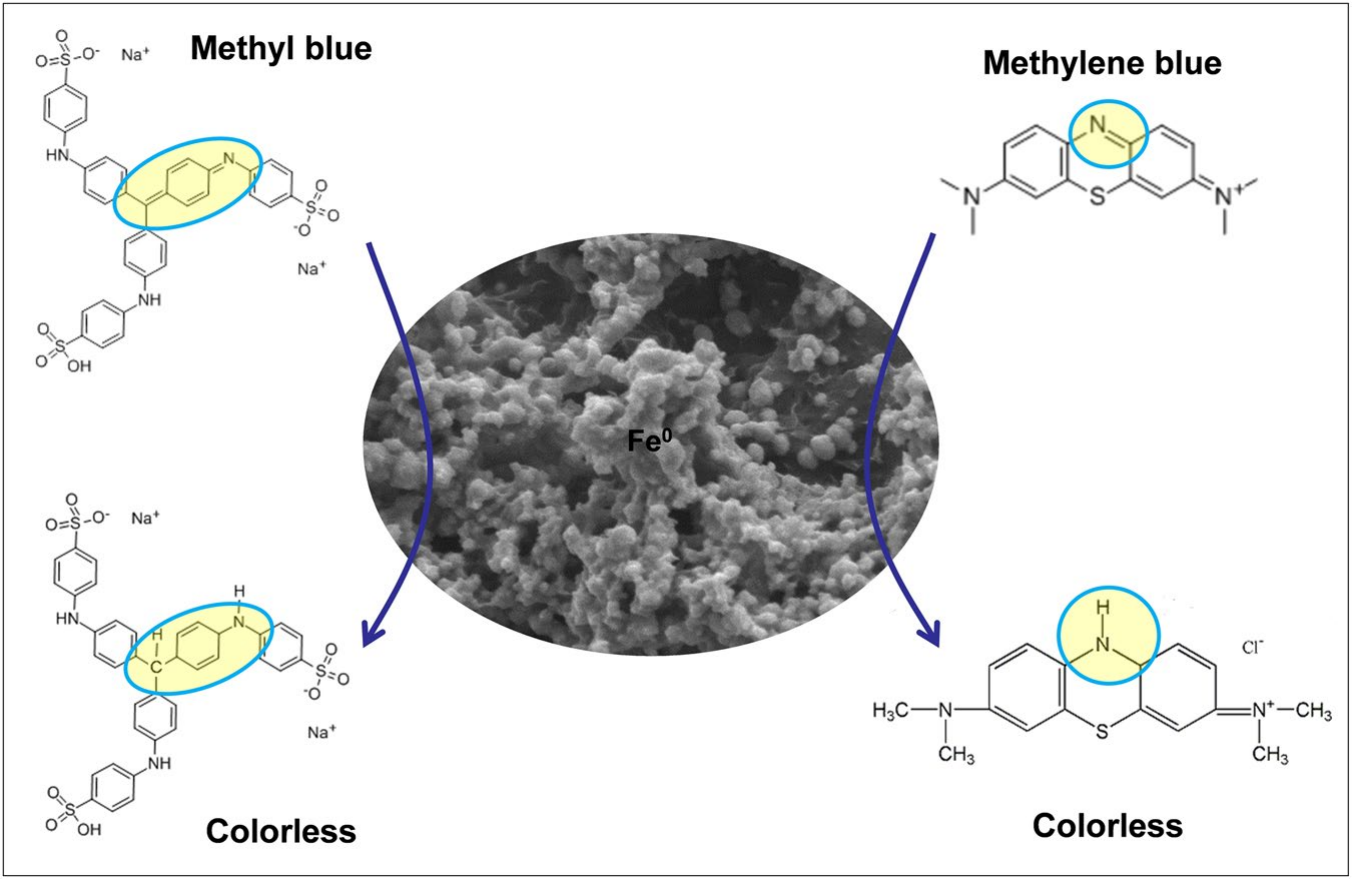
The decoloration mechanism of filter paper supported nZVI (FP/PAA/nZVI).

Methyl blue and methylene blue solutions were selected as the simulated dyeing wastewater (SDW) to evaluate the decoloration efficiency of FP/PAA/nZVI. Figure 4 presents the decoloration of methyl blue with different initial concentration using a piece of FP/PAA/nZVI. The dosage of nZVI was about 0.93 g/L. It showed that all methyl blue solutions were discolored quickly and almost achieved equilibrium in the first 20 minute. After 60 minutes, the decoloration efficiency of methyl blue solution at 10 mg/L, 15 mg/L, 20 mg/L reached 98.3%, 96.8% and 95.2%, respectively. This means that enough adsorption sites were available on FP/PAA/nZVI surface. However, when the solution concentration increased to 25 mg/L and 30 mg/L, the decoloration efficiency decreased to 70.6% and 60.3%. It was because the adsorption sites of FP/PAA/nZVI were saturated as the dye concentration increased. Compared with nZVI particles, FP-supported nZVI prevents aggregation, but decreases the adsorption sites of nZVI to some extent. Figure 5 shows the decoloration of methylene blue solutions using FP/PAA/nZVI, it exhibited similar behavior with methyl blue. Specifically, the methylene blue solutions were also discolored quickly and reached equilibrium in a short time, the decoloration efficiency exceeded 95% for 10 mg/L, 15 mg/L and 20 mg/L methylene blue solutions. Figure 6 presents the digital photographs of 20 mg/L methyl blue and methylene blue solutions with time. Both dye stuff were fully degraded in 25 minutes using FP/PAA/nZVI. Moreover, a consecutive trial was conducted to treat 20 mg/L methylene blue solution using filter flask (Supplementary Movie 1). It showed methylene blue solution fully decolored after three filtrations. As comparison, decoloration of FP/PAA was conducted under the same consecutive trial (Supplementary Movie 2). It clearly showed that color of the methylene blue solution still existed. Besides, it seems that the decoloration efficiency of filtration was higher than that of the beaker shaking method. This may be because with filtration, decoloration occurred on the surface and internal of FP/PAA/nZVI, but the shaking method causes decoloration to occur on the surface. The continuous study paves way for future industrialization. The decoloration mechanism of nZVI has been investigated by some studies. According to Witt’s theory of color constitution, the color of a substance is attributed to the presence of unsaturated cluster referred to as chromophore, which includes -N=N-, -C=C-, -C=N-, -C=O groups and etc. The other groups, like -OH, -COOH and halogen, change and enhance the color of the substance referred to as auxochrome. For methyl blue, the decoloration was attributed to the cleavage of -C=C- and -C=N- by nZVI reduction, which breaks the conjugated structure of dye molecule. For methylene blue, the decoloration was ascribed to the cleavage of -C=N- and -C=S- during the treatment^46^. The decoloration mechanism is shown in Fig. 7.

## Conclusion

In this work, a common filter paper (FP) was modified by *in-situ* polymerization of acrylic acid. nZVI was firmly chelated on the surface and internal of FP by carboxyl group from PAA. The loading content of nZVI in FP reached 24.8%. The preparation approach was simple and environmentally benignant. Most importantly, the fabricated FP/PAA/nZVI exhibited high activity towards the removal of methyl blue and methylene blue. The decoloration efficiency exceeded 95% for 20 mg/L simulated dye solutions after 60 minutes treatment. The decoloration was performed under a filtration process to realize the continuous treatment. We believe that this study paves way for the application of nZVI in the textile industry for the treatment of effluents.

## Supplementary information

Video 1

Video 2
